# Supplementary material for: Intact Mass Profiling Reveals Phospho‐Proteoforms of the Catenins (85–110 kDa) Regulated by Actomyosin Contractility
Source: Angew Chem Int Ed Engl. 2025 Oct 29;64(52):e18593. doi: 10.1002/anie.202518593 (PMC12723464; doi:10.1002/anie.202518593)
Supplement: Supplementary file 1 — Supporting Information [file ANIE-64-e18593-s002.pdf]

SUPPORTING INFORMATION

Intact Mass Profiling Reveals Phospho-Proteoforms of the Catenins (85-110 kDa) Regulated by Actomyosin Contractility

Che-Fan Huang,[a] Taojunfeng Su,[a] Annette S. Flozak,[b] Cara J. Gottardi,*[b] and Neil L. Kelleher*[a]

[a] C.-F. Huang, T. Su, N. L. Kelleher*

Departments of Chemistry, Molecular Biosciences, and Proteomics Center of Excellence

Northwestern University

2170 Campus Dr. Silverman B550, Evanston, Illinois 60208, United States

E-mail: n-kelleher@northwestern.edu

[b] A. S. Flozak, C. J. Gottardi*

Departments of Medicine (Pulmonary and Critical Care), and Cell & Developmental Biology

Feinberg School of Medicine, Northwestern University

303 E. Superior St. Simpson-Querrey 525, Chicago, Illinois 60611, United States

E-mail: c-gottardi@northwestern.edu

*To whom correspondence should be addressed

Table of Contents

Figure S1 I^2MS spectrum of GST-β-catenin S-3
Figure S2 The proteoform landscape of β-catenin S-4
Figure S3 The proteoform landscape of α-catenin S-5
Figure S4 Junctional enrichment of catenins is actomyosin-dependent S-6
Figure S5 LC-MS analysis of cadherin-catenin complexes S-7
Figure S6 Bottom-up analysis of calyculin A-treated Ecad-cyto complexes S-8
Figure S7 β-cat N-terminal sites S-9
Figure S8 I^2MS^2 isolation window of catenins....................S-10
Figure S9 Top-down fragmentation of β-catenin S-11
Figure S10 Top-down fragmentation of α-catenin S-13
Figure S11 Mass error distributions of α- and β-catenins in I^2MS^2 experiments....................S-15
Experimental Section....................S-16
Full-Length Western Blots and Gels....................S-20
Additional References S-22
Table S1 FragPipe Search Results…………………..xlsx

Figure S1

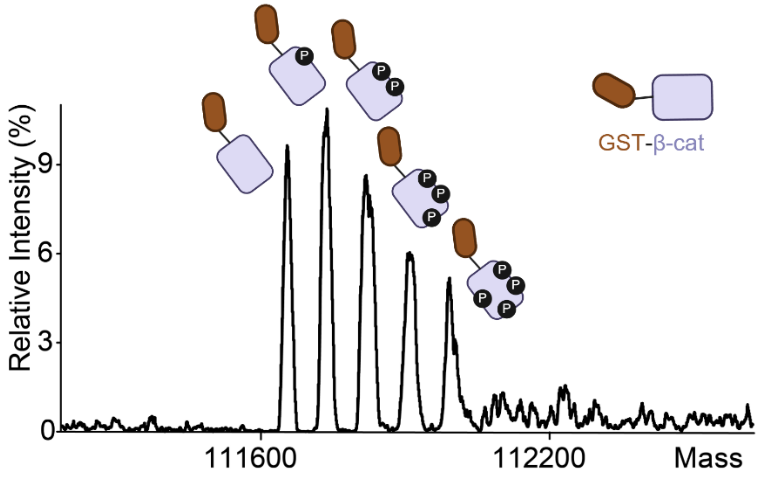

Proteoform	Observed Mass (Da)	Δm to the previous peak (Da)
GST-β-cat (M)	111657.6	-
M+1Phos	111736.9	79.3
M+2Phos	111816.4	79.6
M+3Phos	111897.3	80.9
M+4Phos	111977.5	80.2

Figure S1. I^{2}MS spectrum of GST-β-catenin. Recombinant GST-fused β-catenin (Abcam ab63175, ~112 kDa) was profiled with optimized I^{2}MS parameters in denatured mode, revealing proteoforms separated by 80 ± 1 Da corresponding to phosphorylation. Up to four phosphorylations were observed.

Figure S2

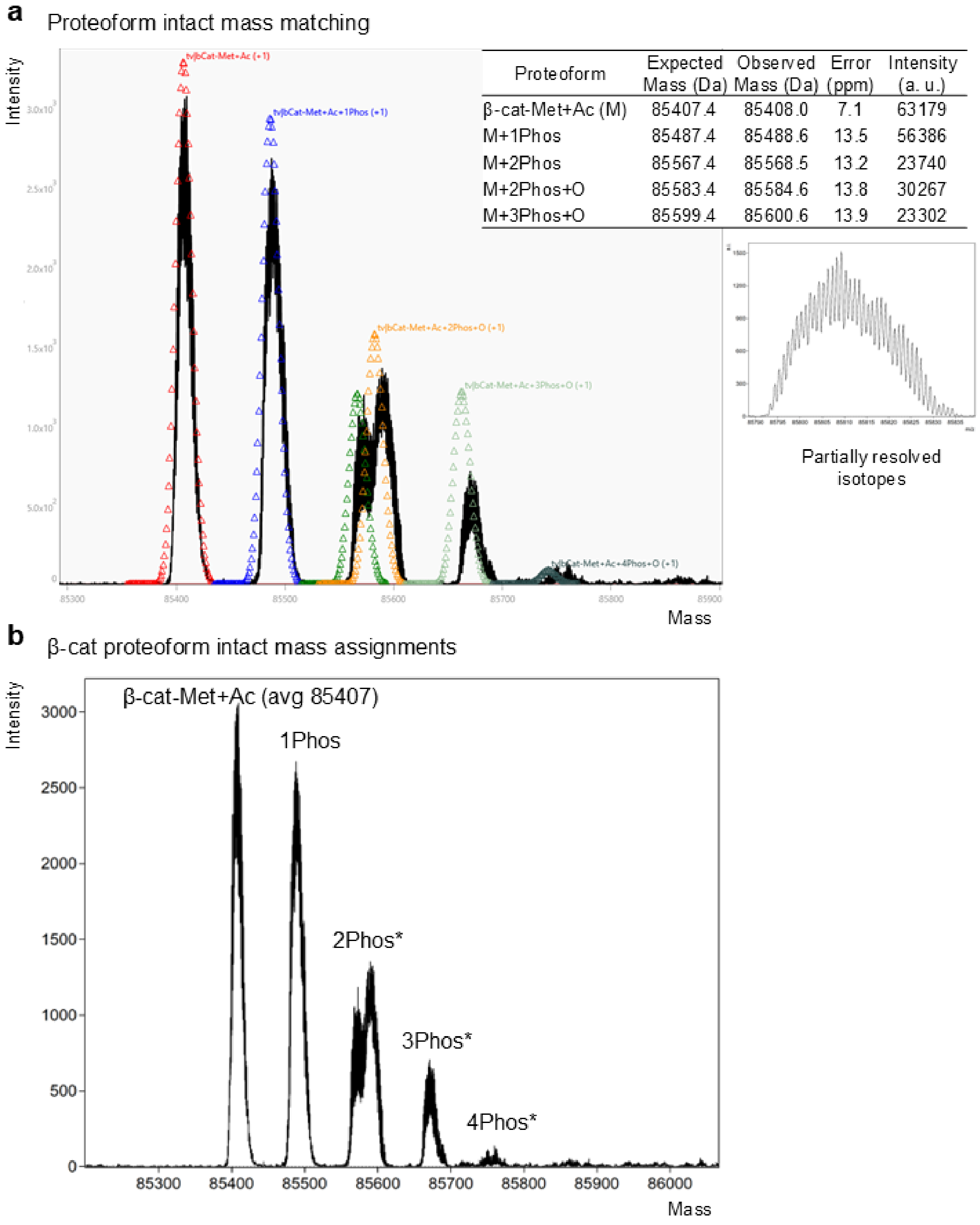

Proteoform	Expected Mass (Da)	Observed Mass (Da)	Error (ppm)	Intensity (a. u.)
β-cat-Met+Ac (M)	85407.4	85408.0	7.1	63179
M+1Phos	85487.4	85488.6	13.5	56386
M+2Phos	85567.4	85568.5	13.2	23740
M+2Phos+O	85583.4	85584.6	13.8	30267
M+3Phos+O	85599.4	85600.6	13.9	23302

Figure S2. The proteoform landscape of β-catenin. (a) Intact mass matching considering phosphorylation and oxidation. Isotopic patterns are partially resolved. (b) β-cat proteoform intact mass assignments. Up to three phosphorylations are observed. *: including oxidized proteoforms.

Figure S3

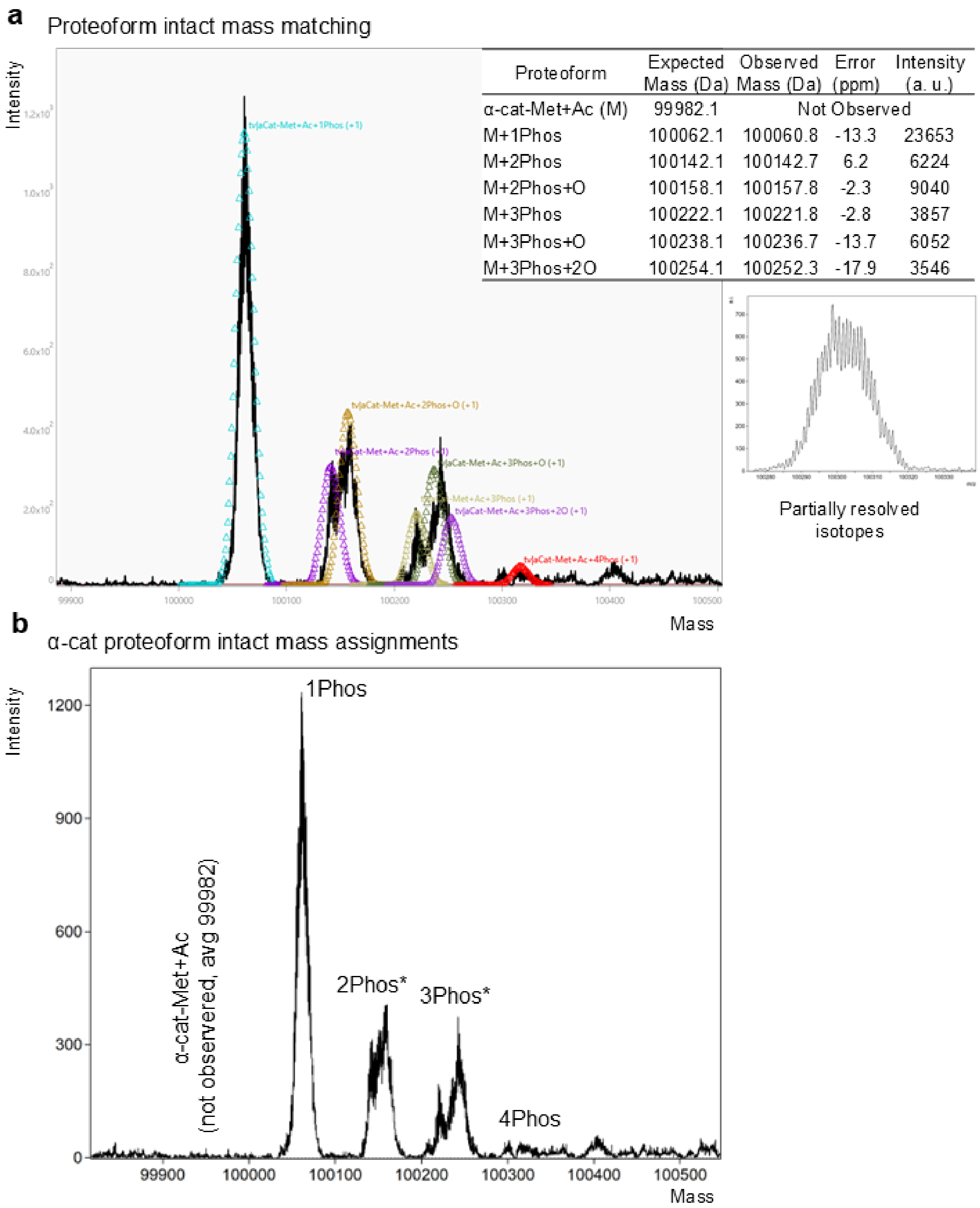

Proteoform	Expected Mass (Da)	Observed Mass (Da)	Error (ppm)	Intensity (a. u.)
α-cat-Met+Ac (M)	99982.1	Not Observed		
M+1Phos	100062.1	100060.8	-13.3	23653
M+2Phos	100142.1	100142.7	6.2	6224
M+2Phos+O	100158.1	100157.8	-2.3	9040
M+3Phos	100222.1	100221.8	-2.8	3857
M+3Phos+O	100238.1	100236.7	-13.7	6052
M+3Phos+2O	100254.1	100252.3	-17.9	3546

Figure S3. The proteoform landscape of α-catenin. (a) Intact mass matching considering phosphorylation and oxidation. Isotopic patterns are partially resolved. (b) α-cat proteoform intact mass assignments. There is no unphosphorylated α-cat. There is no unphosphorylated α-cat detected in the cadherin complex. One to three phosphorylations are observed. *: including oxidized proteoforms.

Figure S4

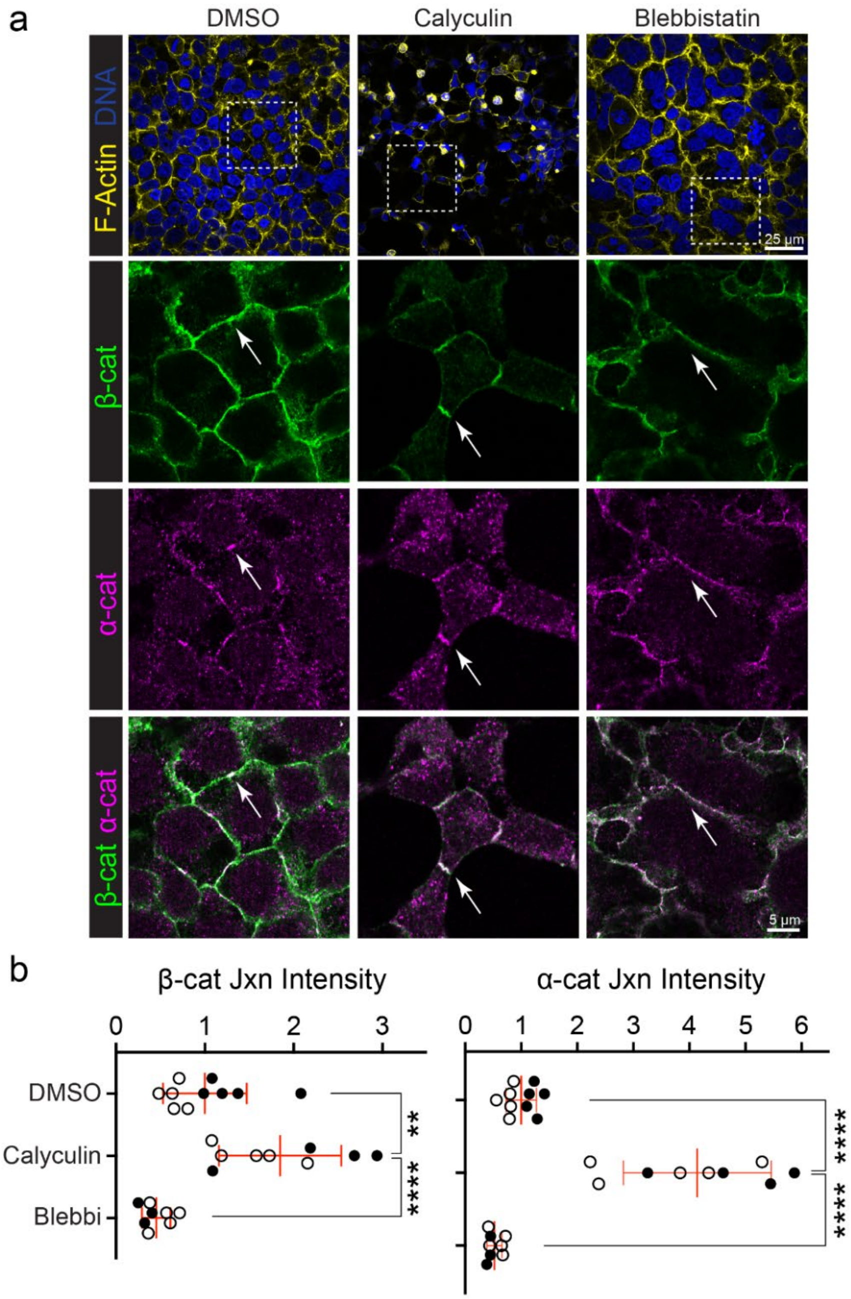

Figure S4. Junctional enrichment of catenins is actomyosin-dependent. (a) HEK cells treated with DMSO (control), calyculin (actomyosin activator), and blebbistatin (actomyosin inhibitor) were stained for DNA (blue), F-actin (yellow), β-cat (green) and α-cat (magenta) under a fluorescent microscope. Arrow: adherens junction. (b) Graphs show β-cat and α-cat fluorescence intensities at the junction (Jxn minus extra-junctional cytoplasmic intensity) under different actomyosin-perturbing conditions. Calyculin increased catenin junction enrichment whereas blebbistatin decreased enrichment. Error bars: one standard deviation. **: $P<0.01$; ****: $P<0.0001$ (Student's *t*-test).

Figure S5

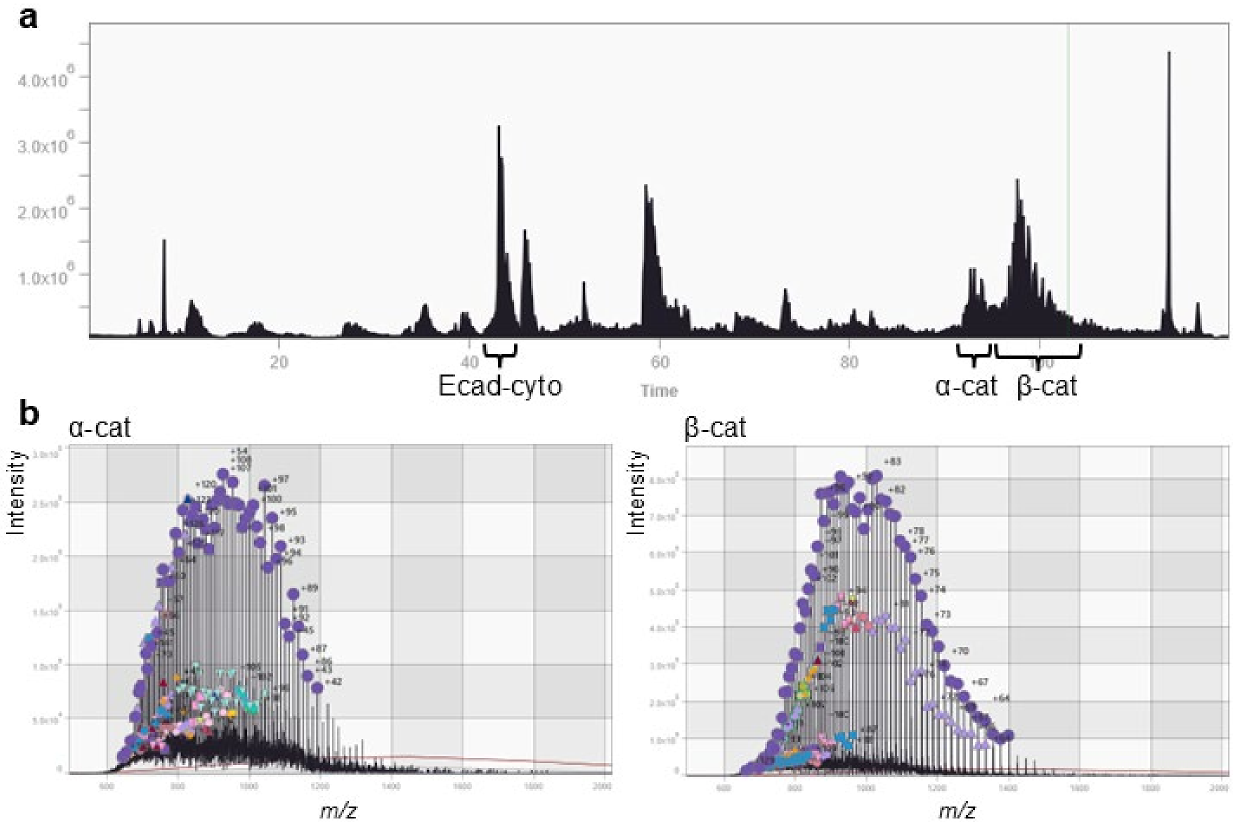

Figure S5. LC-MS analysis of cadherin-catenin complexes. (a) Chromatogram of Ecad-cyto eluate analyzed over a 2 h gradient. (b) Ensemble MS is low resolution for highly charged catenins, resulting in extensive spectral congestion of proteoforms.

Figure S6

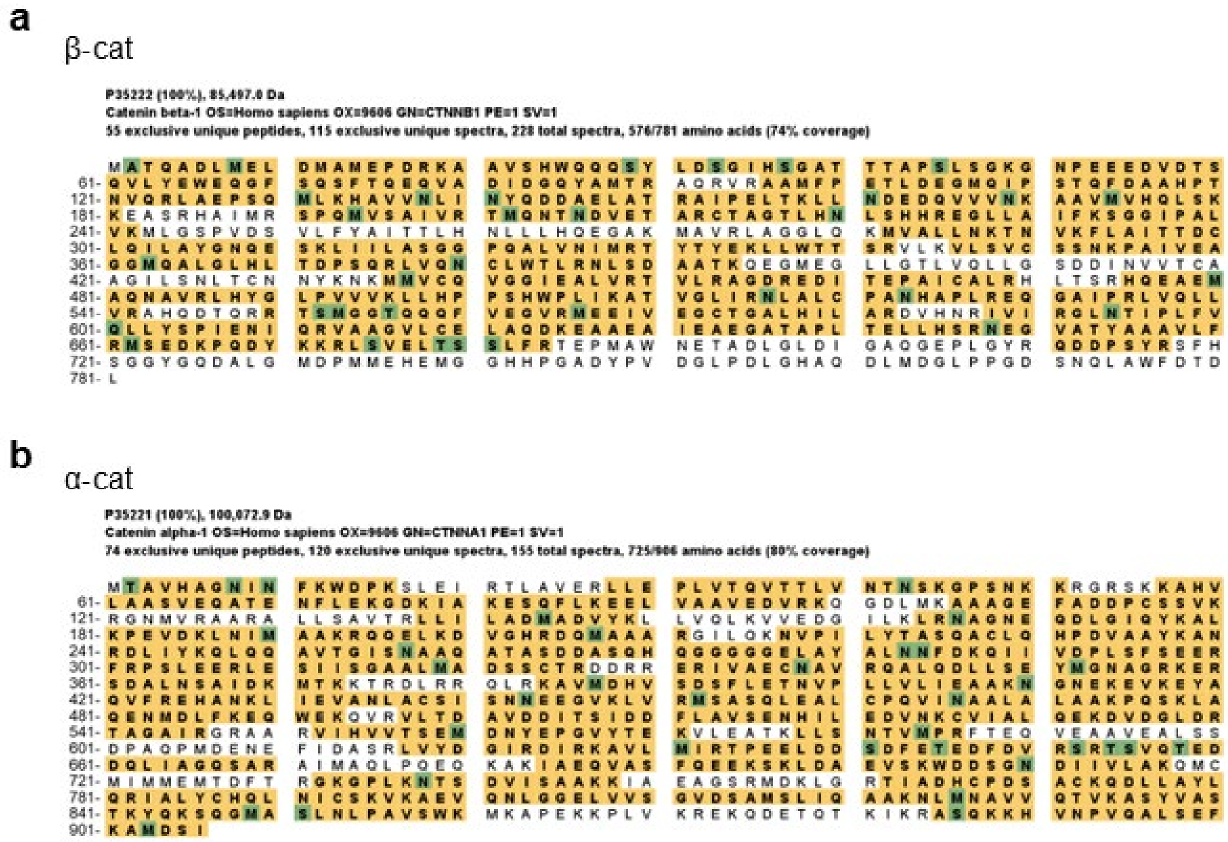

Figure S6. Bottom-up analysis of calyculin A-treated Ecad-cyto complexes. (a) Sequence coverage map of β-cat. (b) Sequence coverage map of α-cat. Peptide search was performed on MASCOT including variable phosphorylation at S, T, Y residues and the results were visualized with Scaffold software. Yellow: identified peptides; green: modified residues (including ambiguous modifications).

Figure S7

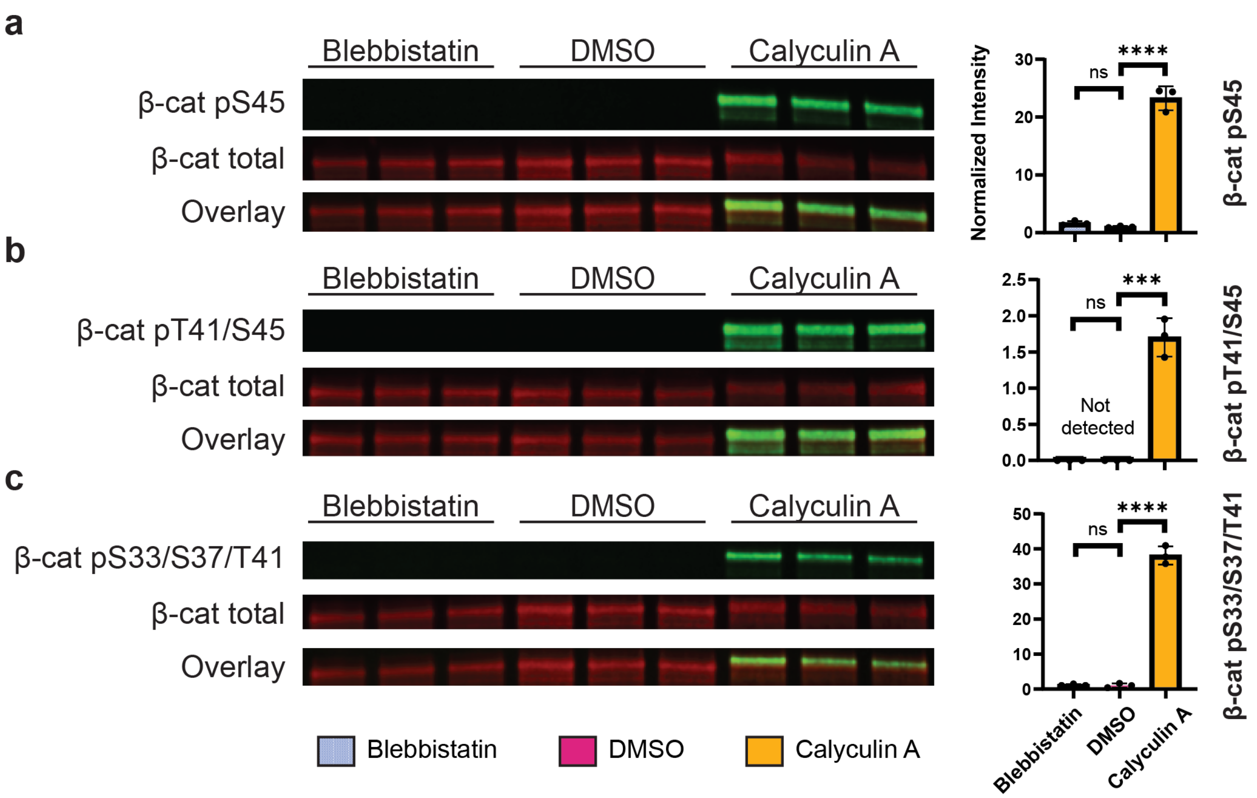

Figure S7. β-cat N-terminal sites. (a-c) pS45, pT41, pS37 and pS33 are only detected in HEK cells treated with calyculin A. These sites are traditionally associated with GSK3β-mediated degradation pathways. All experiments were performed in triplicates. Intensities of catenin phospho-antibodies were divided by corresponding total catenin intensities and normalized to the ratio in DMSO controls. Error bars: one standard deviation. ns: $P>0.05$; *: $P<0.05$; **: $P<0.01$; ***: $P<0.001$; ****: $P<0.0001$ (Student's *t*-test, n = 3).

Figure S8

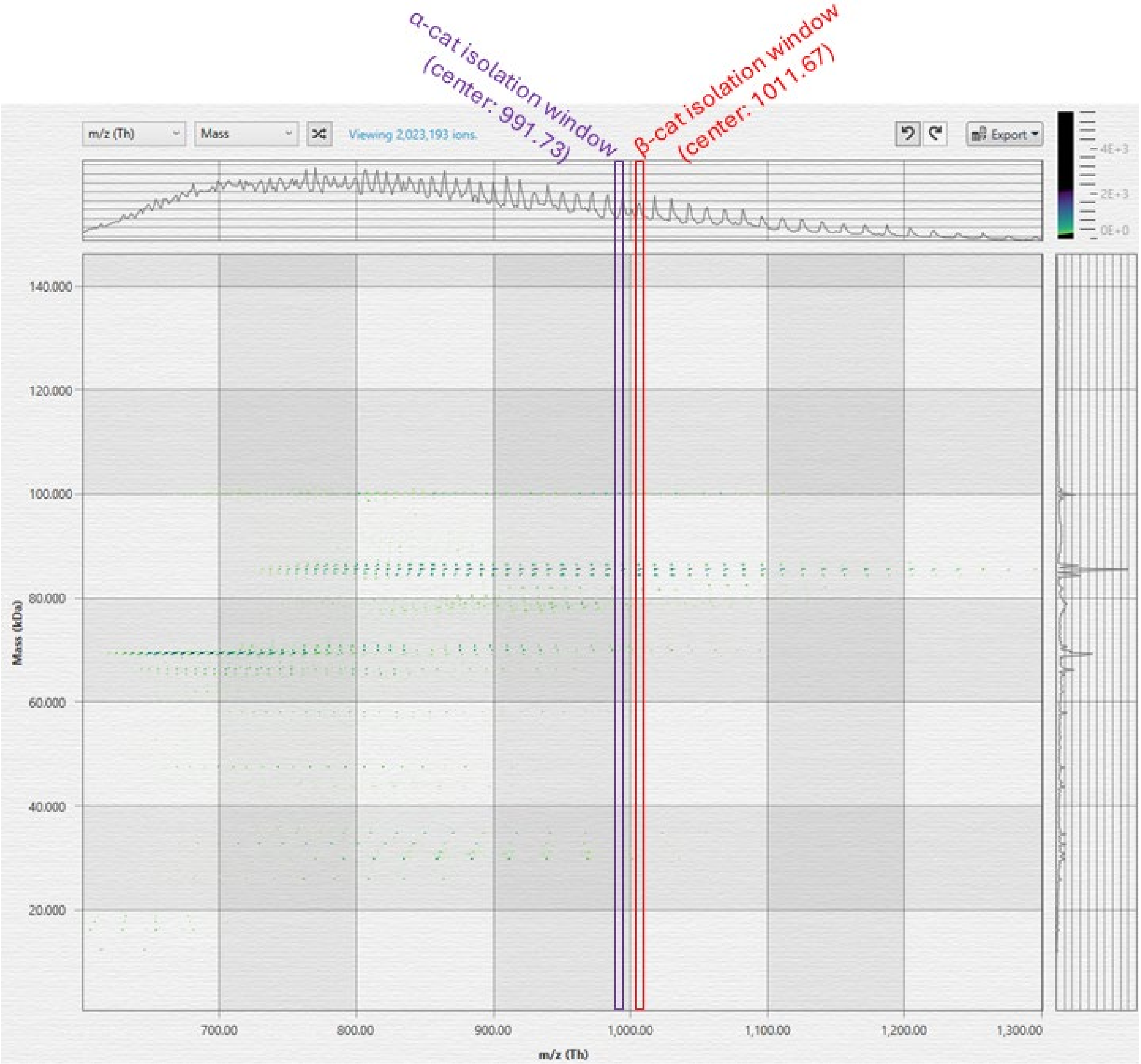

Figure S8. I^2MS^2 isolation window of catenins. β-catenin (red) and α-catenin (purple) were isolated using a quadrupole (2 Th window) to isolate their full proteoform landscape in a single charge state (centers of *m/z*: β-cat 1011.67, 85+ charge state; α-cat 991.73, 101+ charge state) for fragmentation and I^2MS^2 detection.

Figure S9

a β-cat I²MS² fragmentation

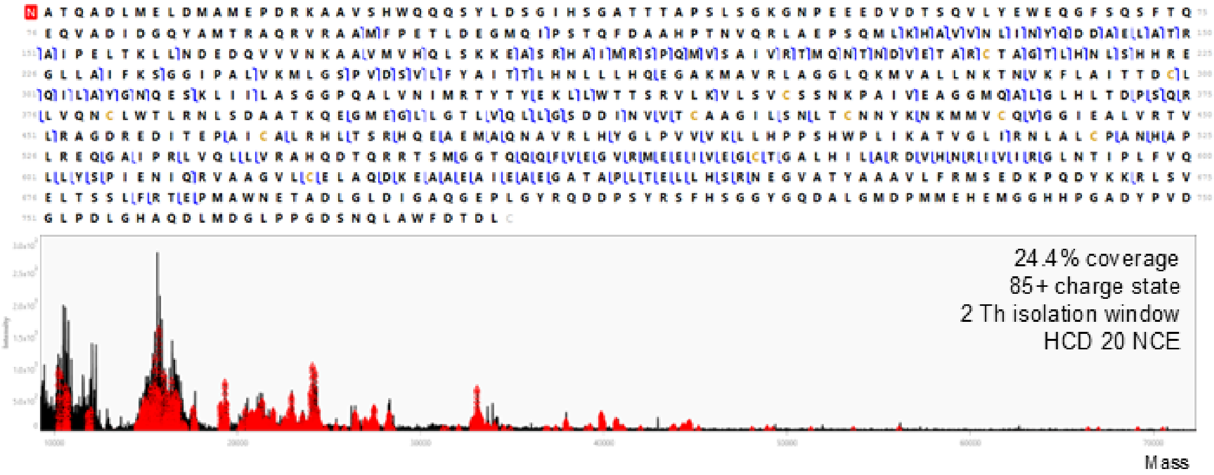

b β-cat ensemble LC-MS/MS fragmentation (HCD)

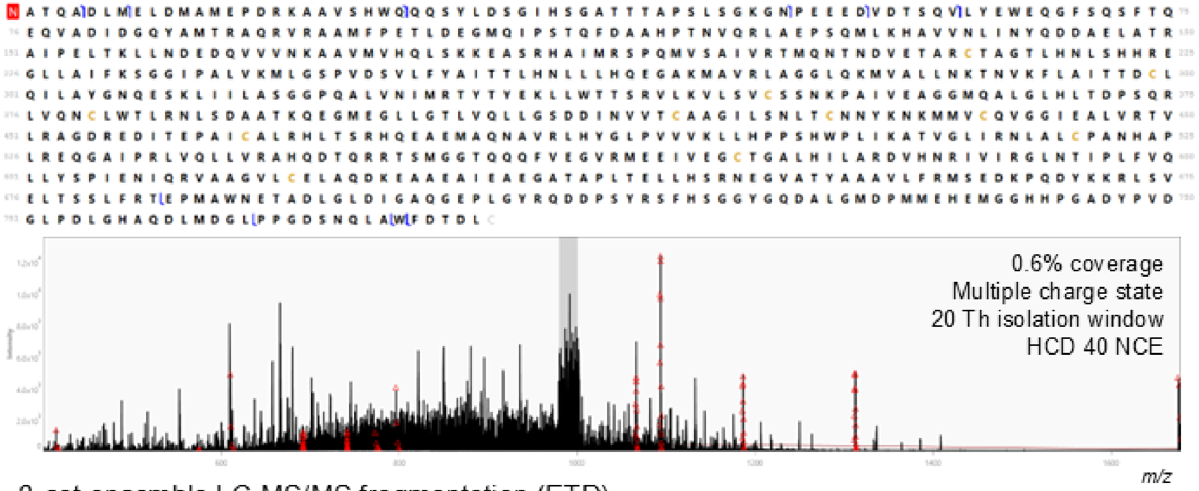

c β-cat ensemble LC-MS/MS fragmentation (ETD)

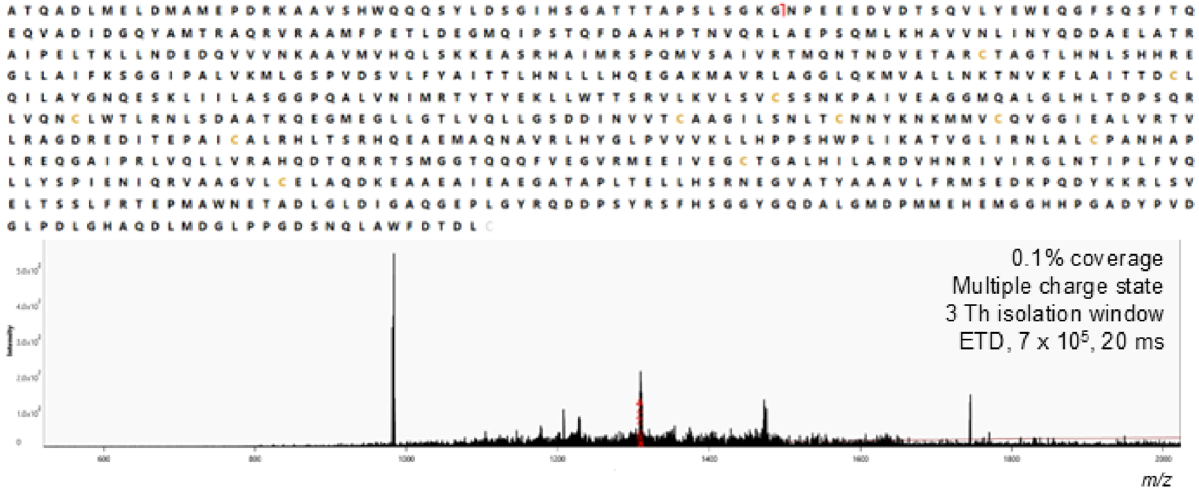

Figure S9. Top-down fragmentation of β-catenin. (a) β-cat fragment ion detection was significantly improved by individual ion mass spectrometry for MS^2-level experiments. (b-c) Representative β-cat LC-MS/MS spectrum and sequence coverage map acquired using traditional ensemble detection method combined with HCD (b) and ETD (c) fragmentation.

Figure S10

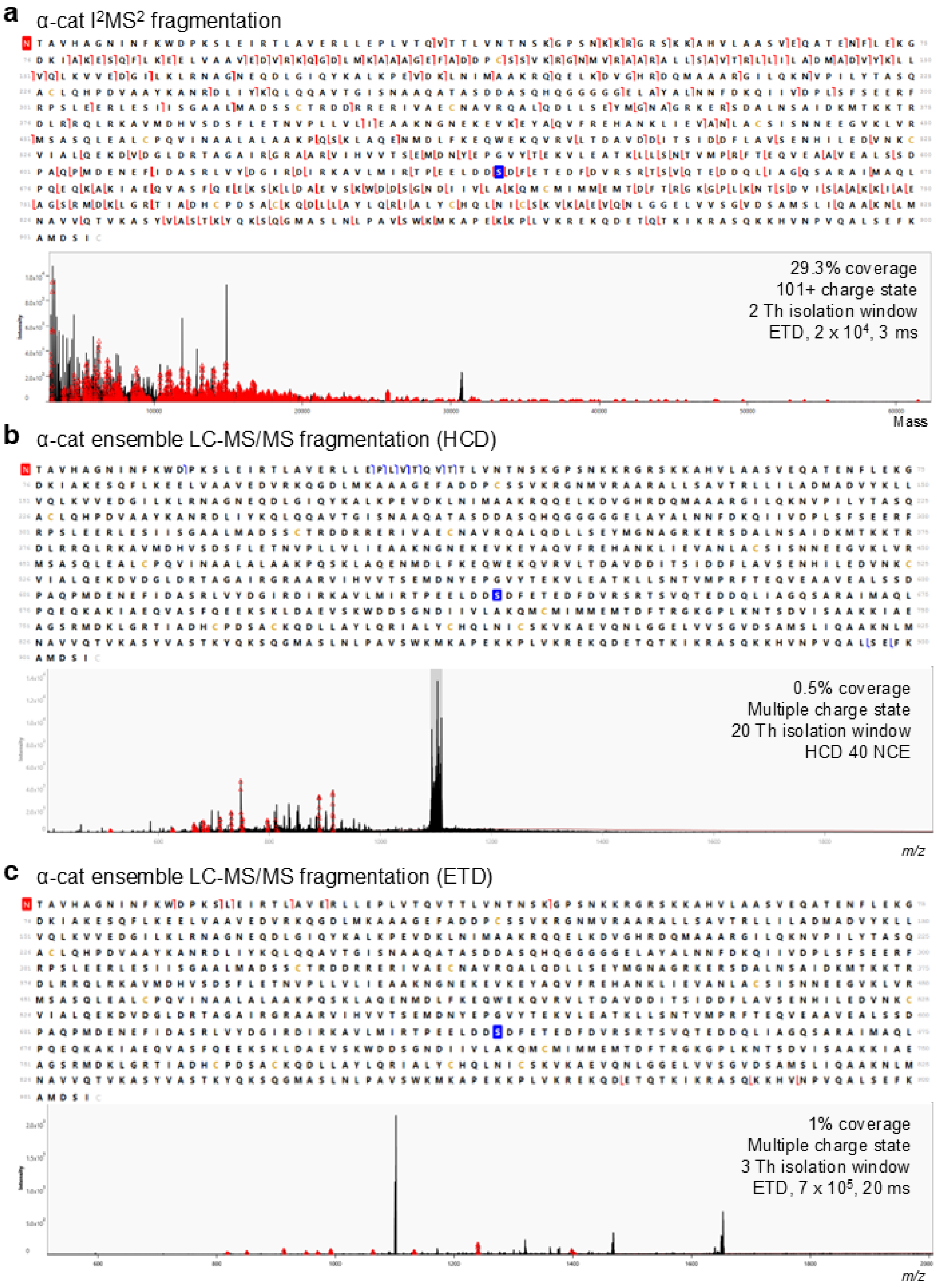
a
α-cat I²MS² fragmentation
29.3% coverage
101+ charge state
2 Th isolation window
ETD, 2 x 10⁴, 3 ms
Mass
b
α-cat ensemble LC-MS/MS fragmentation (HCD)
0.5% coverage
Multiple charge state
20 Th isolation window
HCD 40 NCE
m/z
c
α-cat ensemble LC-MS/MS fragmentation (ETD)
1% coverage
Multiple charge state
3 Th isolation window
ETD, 7 x 10⁵, 20 ms
m/z

Figure S10. Top-down fragmentation of α-catenin. (a) α-cat fragment ion detection was significantly improved by individual ion mass spectrometry for MS^2-level experiments. (b-c) Representative α-cat LC-MS/MS spectrum and sequence coverage map acquired using traditional ensemble detection method combined with HCD (b) and ETD (c) fragmentation.

Figure S11

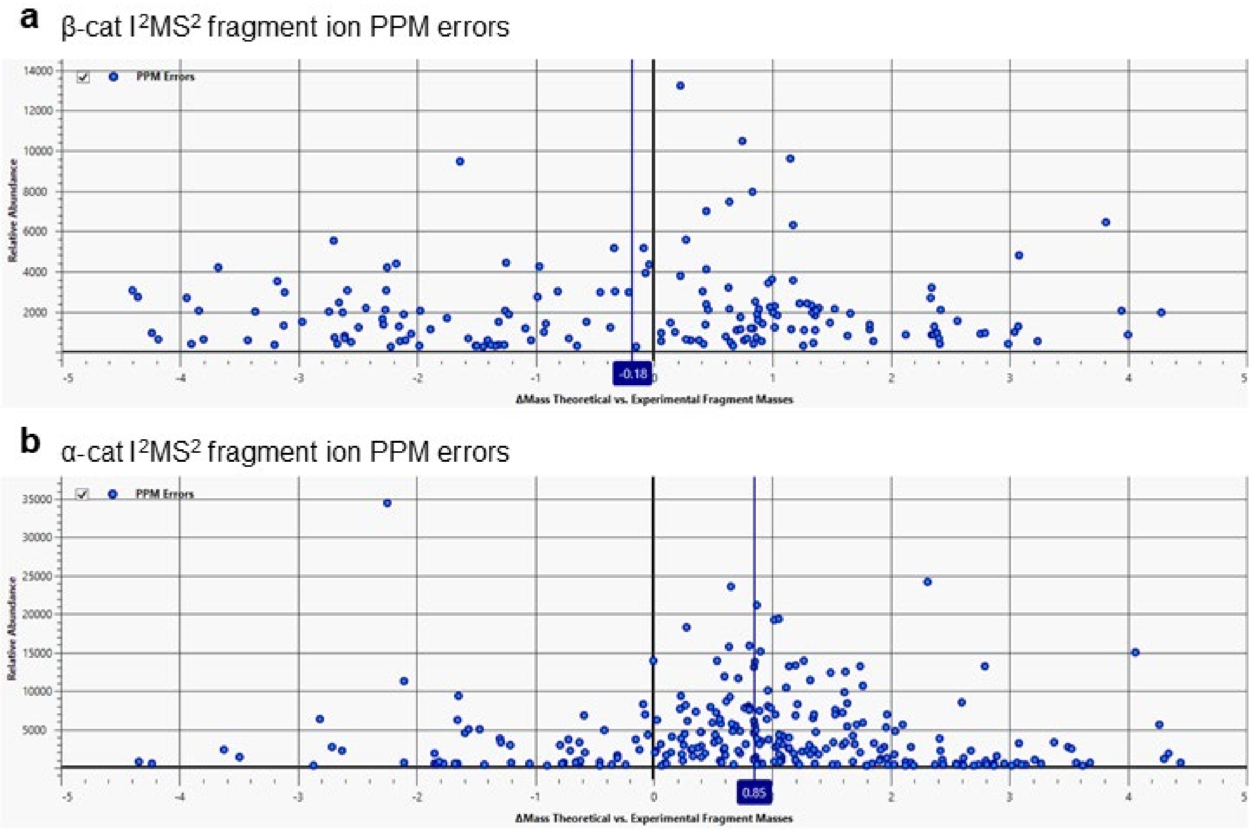

Figure S11. Mass error distributions of α- and β-catenins in I^2MS^2 experiments. A max mass tolerance of ±5 ppm was applied to the identification of α- and β-catenin fragment ions in TDValidator software.

Experimental Section

Antibodies. The following primary antibodies were used in this study for western blots: mouse anti-β-catenin (610153, BD Bioscience), mouse anti-α-catenin (ALX-804-101, Enzo), rabbit anti-α-catenin (3236, Cell Signaling), mouse anti-c-myc (M4439, Millipore Sigma), rabbit anti-β-catenin pS33/S37/T41 (9561, Cell Signaling), rabbit anti-β-catenin pT41/S45 (9565, Cell Signaling), rabbit anti-β-catenin pS45 (9564, Cell Signaling), rabbit anti-β-catenin pS552 (9566, Cell Signaling), rabbit anti-β-catenin pS657 (9567, Cell Signaling), rabbit anti-α-catenin pS641 (11330, Signalway), rabbit anti-α-catenin pS652 (13061, Cell Signaling), and rabbit anti-α-catenin pS655/T658 (13231, Cell Signaling). The following primary antibodies were used in this study for immunofluorescence and microscopy: mouse anti-β-catenin (610153, BD Bioscience), rabbit anti-α-catenin (3236, Cell Signaling), Alexa Fluor 568 phalloidin (A12380, Invitrogen), Alexa Fluor 647 phalloidin (A22287, Invitrogen). The secondary antibodies were: IRDye 680 RD donkey anti-mouse (926-68072, LI-COR, western blot), IRDye 800 CW donkey anti-rabbit (926-32213, LI-COR, western blot), Alexa Fluor 488 goat anti-mouse (A-11001, Invitrogen, microscopy), and Alexa Fluor 568 goat anti-rabbit (A-11001, Invitrogen, microscopy).

Cell culture. HEK 293T cells were obtained from American Type Culture Collection (ATCC). The Ecad-cyto HEK 293T cell line was previously created and validated.[1] The plasmid sequence was recently checked with Sanger sequencing for authentication. Cells were maintained in Dulbecco's Modified Eagle's Medium (DMEM, Corning), containing 10% fetal bovine serum (FBS, Atlanta Biologicals), 100 U/ml penicillin and 100 mg/mL streptomycin (Corning) at 37°C and 5% CO_2. Ecad-cyto HEK cells were supplemented with 0.25 mg/mL Hygromycin B (Gibco). For actomyosin treatments, blebbistatin (10 mM, Millipore Sigma) and calyculin A (10 μM, Millipore Sigma) stock solutions were prepared in sterile DMSO. Cells were treated with blebbistatin (20 μM, 24 h), DMSO (control, 0.2%, 24 h) or calyculin A (20 nM, 2 h) in fresh media.

Confocal microscopy. Microscope cover glasses (22 x 22 mm, #1.5, Fisher Scientific) were flamed and coated with 1 mL poly-D-lysine (0.1 mg/mL, Gibco) in a 12 well plate for 1 h at room temperature. HEK cells were seeded at a seeding density of 2.5 x 10^4 cells and incubated for 4 days and treated with blebbistatin, DMSO or calyculin A in fresh media. Cells were fixed in 4% paraformaldehyde (PFA) in PBS for 15 min, quenched with 100 mM glycine, permeabilized with 0.3% Triton X-100, and blocked with 3% normal goat serum (Millipore Sigma). Primary (1:100) antibodies were incubated at room temperature for 1 h and secondary antibody (1:300) incubations were performed at room temperature for 30 min Phalloidin (1:50) was added to the secondary antibody incubation for F-actin staining. Hoechst (1:10,000, Thermo Fisher) was incubated for 5 min, interspaced by multiple washes in PBS, and followed by mounting coverslips in ProLong Gold fixative (Life Technologies). Fixed cells were imaged on a Nikon A1 (CAM A1RB) microscope with GaAsP detectors and equipped with a 95B prime Photometrics camera using S Fluor 40x Oil DIC H N2 objective or APO 60x I Oil objective. Confocal *z*-stacks were collected at 0.25 μm step size. Captured images were processed in Fiji software. β- and α-catenin intensities were quantified at cell-cell junctions and statistical analyses were performed in GraphPad Prism.

Cell lysis and immunoprecipitation. Ecad-cyto HEK cells were grown to confluency in a 150 mm dish (Corning), treated with blebbistatin, DMSO or calyculin A in fresh media, washed with PBS and harvested in cell lysis buffer [50 mM Tris, 150 mM NaCl, 5 mM EDTA, 1% Triton X100, pH 7.5 and 1X Halt protease/phosphatase inhibitor cocktail (Thermo Fisher Scientific)]. Cell lysate was sonicated and centrifuged at 14,000 rpm for 10 min to remove debris. The concentration was determined by Bradford assay (5000006, Bio-Rad). Lysate was incubated overnight with 25 µL myc-trap agarose (ChromoTek) at 4°C under agitation. Immunocomplexes were collected by centrifugation and washed once with ice-cold cell lysis buffer, twice with 10X TBS (Fisher Scientific), twice with 1X TBS and once with LC-MS grade water (Fisher Scientific). For TDP experiments, Ecad-cyto complexes were eluted with 100 µL 1% formic acid (LC-MS grade, Fisher Scientific) at 37°C for 10 min For BUP and electrophoresis experiments, Ecad-cyto complexes were eluted with 100 µL 2X SDS-PAGE sample buffer (Bio-Rad) containing 5% β-mercaptoethanol (Sigma-Aldrich) at 95°C for 10 min.

Electrophoresis and immunoblotting. Lysates or IP eluates were resolved by 4-20% Criterion TGX Midi gels (Bio-Rad, 150 V, 90 min) and transferred to nitrocellulose membranes using the Trans-Blot Turbo system (Bio-Rad) following the manufacturer's protocol. Membranes were blocked in Intercept (TBS) blocking buffer (LI-COR) for 1 h at room temperature, incubated overnight with primary antibodies at 4°C and then with fluorophore-conjugated secondary antibodies (LI-COR) for 1 h at room temperature. All antibodies were used at the dilutions suggested by the manufacturers. The bands were visualized by LI-COR Odyssey Fc Imaging System. The images were processed with ImageJ and statistical analyses were performed in GraphPad Prism.

Individual ion mass spectrometry. IP eluates were buffer exchanged into electrospray-compatible system buffer (69.8% water, 30% acetonitrile, 0.2% formic acid) using the SampleStream platform.[2] Direct injection and I^{2}MS data acquisition was automated using SampleStream. Buffer exchanged sample (70 µL) was picked up by a PAL3 robot, delivered to an Q Exactive HF mass spectrometer (Thermo Fisher Scientific) at a flow rate of 1.2 µL/min, and sprayed through a customed nanospray source. Upon sample injection, a process coined Automated Ion Control (AIC) attenuated ensemble ion signals down to an ion injection time (1–400 ms) corresponding to optimal individual ion collection on a per-sample basis to maximize the number of individual ion signals without producing a large number of multiple ion events at singular *m/z* values.[3] Other instrument parameters included: eFT, off; Orbitrap central electrode voltage, 1 kV; trapping gas pressure, 0.5; spray voltage, 2.6 kV; sheath gas, 0 L/min; in-source CID, 15 eV; source temperature, 320°C; *m/z* acquisition range, 500–3000 *m/z*; resolution 45,000 @ 200 *m/z* (1 s transient time); the data was collected for 1 h. I^{2}MS^2 data acquisition was performed using manual direct injection via a syringe pump (1 µL/min) on an Orbitrap Eclipse mass spectrometer (Thermo Fisher Scientific). Precursor ions were selected using a quadrupole with a 2 Th isolation window (centers of *m/z*: β-cat 1011.67, α-cat 991.73). HCD (NCE 20 @ 85+ charge state) was used for β-cat fragmentation and ETD (reagent density 2 x 10^4, 3 ms reaction time) was used for α-cat. The Orbitrap resolution was 240,000 @ 200 *m/z* (2 s transient time), and the scan range was 400–2,000 *m/z*. The injection time was manually optimized for maximum single ion events (typically 10-50 ms), and the normalized AGC target was 500%. The ion routing multipole (IRM) pressure was

set to 0.001 Torr to extend the survival of large fragment ions in I^2MS^2 readout. The I^2MS data were acquired for 1 h.

Data processing. All of the .storx files collected from I^2MS and I^2MS^2 experiments were processed using STORIboard (https://www.proteinaceous.net/storiboard).[4] Charges were assigned with the voting charge assignment algorithm. Based on the ion charge and *m/z*, the accurate mass of each ion was calculated, and a spectrum with all of the charge-assigned ions presenting in the mass domain was generated in a *.mzML* file. Further annotation of I^2MS and I^2MS^2 data was performed using TDValidator (Proteinaceous, https://www.proteinaceous.net/tdvalidator).[5] For I^2MS, the catenin proteoforms were assigned to spectral peaks according to the matches of intact masses at a maximum tolerance of ±20 ppm and a mass tolerance of 1 Da. For I^2MS^2 data, matches to *b*, *y*, *c* and *z* fragment ions from a specific catenin proteoform were annotated with the following settings: a max tolerance of ±5 ppm (**Figure S11**); additional settings in TDValidator were a minimum score of 0.5 (between 0-1, indicating the overall similarity between theoretical and experimental ion clusters) and S/N of 3. A step-by-step TDValidator tutorial can be found in previously published work.[5]

Top-down LC-MS/MS. Proteins in the IP eluate were separated using a Vanquish Neo UHPLC chromatographic system (Thermo Fisher). Reversed-phase LC was performed on a MAbPac EASY-Spray column (150 mm length by 150 µm inner diameter, Thermo Fisher Scientific) with an in-house packed PLRP-S trap (25 mm length by 150 µm i.d., Agilent). The total run time was 120 min using a gradient of mobile phase A (99.9% water and 0.1% formic acid) and mobile phase B (19.9% water, 80% acetonitrile, and 0.1% formic acid). The flow rate was set at 1 µL/min, and the gradient used to resolve proteins was 5% B at 0 min, 30% B at 5 min, 55% B at 110 min, 99% B from 111 to 114 min, and 5% B from 115 to 120 min. The column outlet was coupled inline to an EASY-Spray source and an Orbitrap Eclipse or Ascend mass spectrometer (Thermo Fisher Scientific) operating in intact protein mode with 2 mTorr of N_2 pressure in the ion routing multiple (IRM). The transfer capillary temperature was set at 320°C, the ion funnel RF was set at 60%, and 15 V of source CID was applied. MS^1 spectra were acquired at 7,500 resolving power (at *m/z* 200), a normalized AGC target of 1000%, 1200 ms maximum injection time, and 16 µscan. MS^2 spectra were acquired in proteoform reaction monitoring (PfRM) mode.[6] Spectra were acquired at 60,000 resolving power (at *m/z* 200), normalized AGC target value of 1200%, 1000 ms of maximum injection time, and 4 µscan/spectrum. Higher-energy collisional dissociation (HCD) was used with the normalized collision energy (NCE) set at 40% to generate MS^2 fragmentation spectra. Precursors were quadrupole-isolated by using a 20 Th wide isolation window. The following precursor *m/z* isolation window centers were applied: β-cat 990 *m/z*, α-cat 1100 *m/z*.

Bottom-up proteomics analysis. Bottom-up proteomics was performed at Northwestern Proteomics Core Facility. Calyculin A-treated HEK cell Ecad-cyto eluate was digested with trypsin (Promega) following the manufacturer's in-gel digestion protocol. LC-MS/MS analysis was performed on a Vanquish Neo UHPLC (Thermo Fisher Scientific) coupled to an Orbitrap Exploris 240 mass spectrometer (Thermo Fisher Scientific). Analytical separation was conducted using a UHPLC C18 column (Ion Opticks, AUR3-15075C18-CSI). The flow rate was set at 0.2 µL/min Elution of peptides from analytical separation column was performed using a 120 min gradient between buffer A (0.1% formic acid and

99.9% Optima LC/MS grade water) and buffer B (80% acetonitrile, 19.9% Optima LC/MS grade water, and 0.1% formic acid): 0% B at the beginning, 8% B at 1 min, 28% B at 86 min, 50% B at 106 min, 100% B from 107 to 110 min, 0% B from 111 to 120 min. Electrospray ionization using Orbitrap Exploris 240 was performed using a Nanospray Flex Ion Source (Thermo Fisher Scientific) and positive static spray voltage was set at 2400 V. For full MS^1, scan range was set to 350-1600 *m/z*. The instrument is set in peptide mode, RF lens: 60%, Orbitrap resolution: 120,000, Normalized AGC Target (%): 300, Maximum injection time (ms): 25, Microscans: 1, and Intensity threshold: 5.0e3. Data-dependent acquisition (DDA) by TopN was performed through fragmentation isolated precursor ions with charges between +2 to +5. The following parameters were used for DDA: Dynamic exclusion mode: exclusion duration (s): 30, mass tolerance: 5 ppm (low) and 5 ppm (high). Data dependent mode: cycle time (s): 2, ddMS2 parameters isolation window (*m/z*): 1.5, Normalized collisional energy: 30%, Orbitrap resolution 15,000, scan range mode: define first mass, first mass (*m/z*): 200, Normalized AGC target (%): 100, Maximum injection time (ms): 50, Microscan: 1. The data was search against the human FASTA database (UniProtProteome: UP000005640) from UniProt using MASCOT[7] and FragPipe with MSFragger for PTM analysis.[8]

Data availability. All mass spectrometry *.raw* data, I2MS STORI files, *.mzML* spectra are available on MassIVE through repository number MSV000098690. There is no password needed to access the files.

Full-Length Western Blots and Gels

Fig. 2c

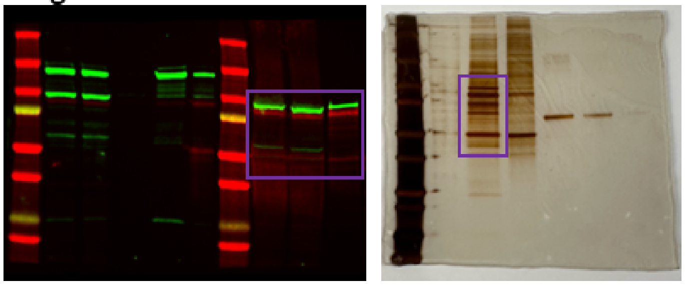

Fig. 4a

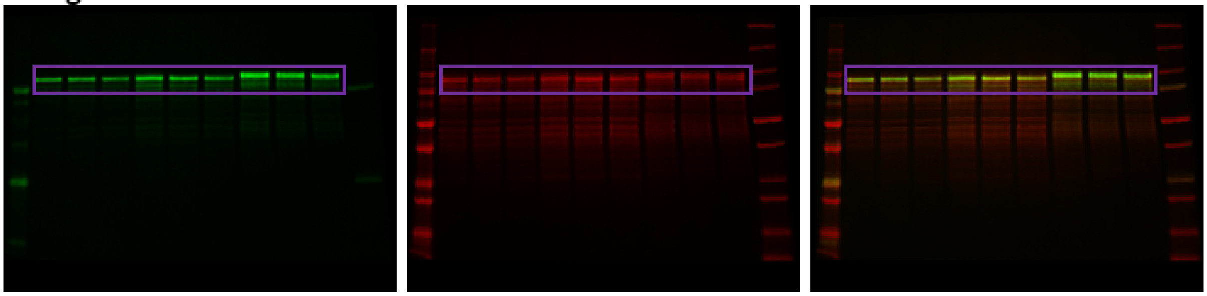

Fig. 4b

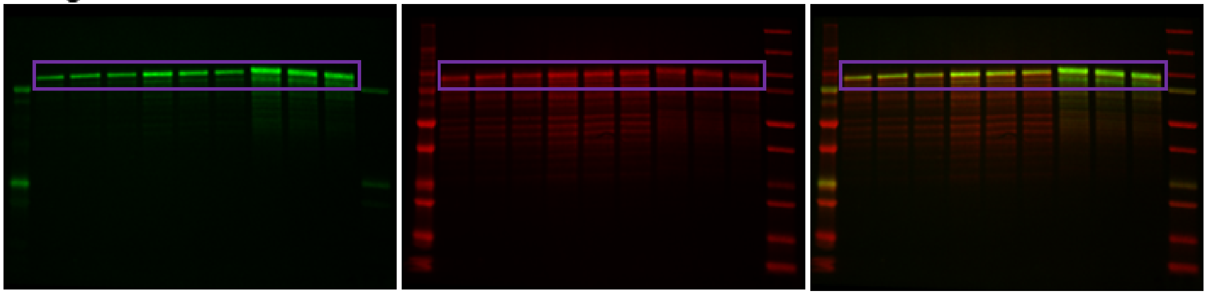

Fig. 4c

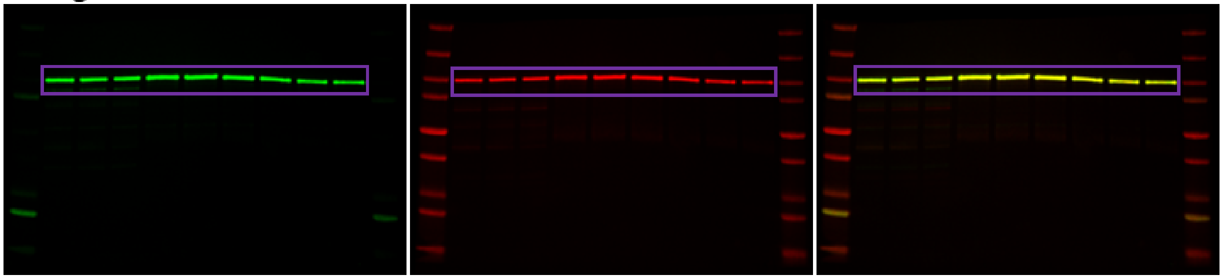

Fig. 4d

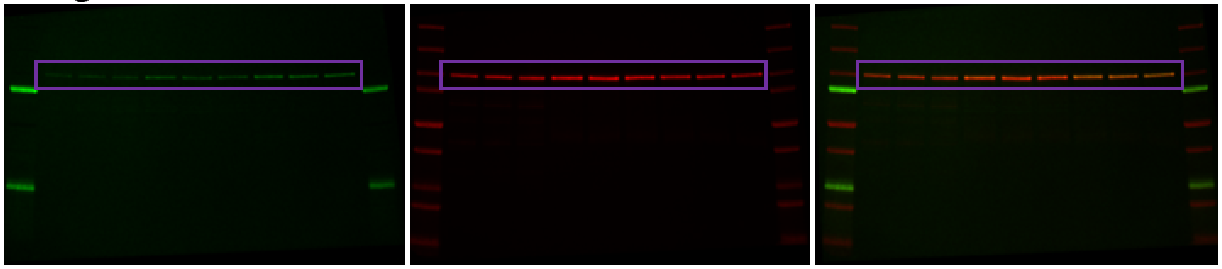

Fig. 4e

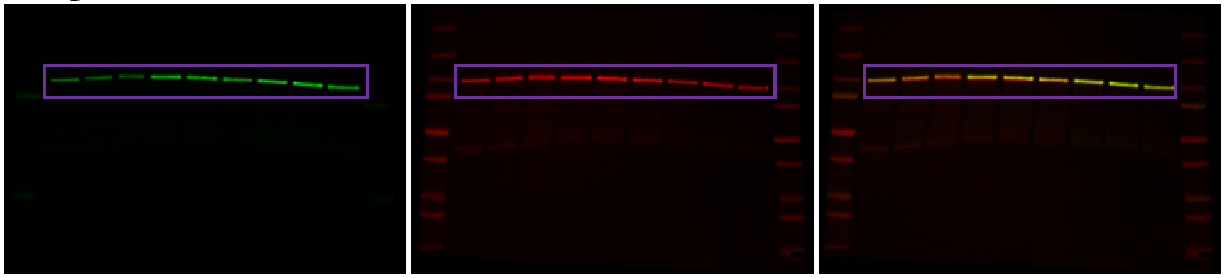

Fig. S7a

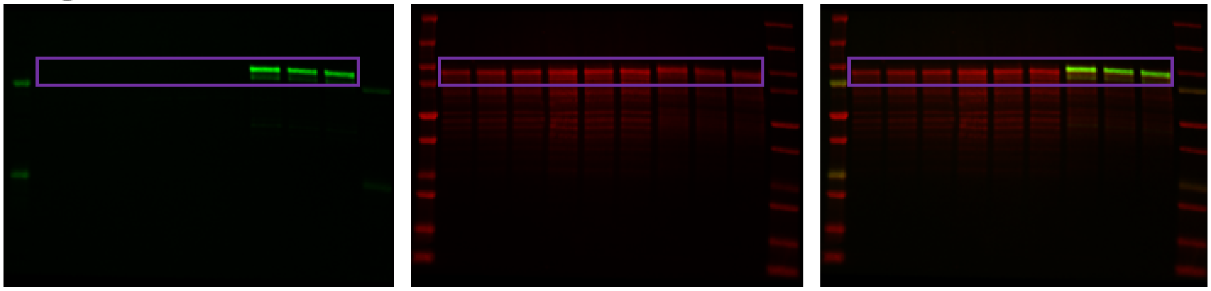

Fig. S7b

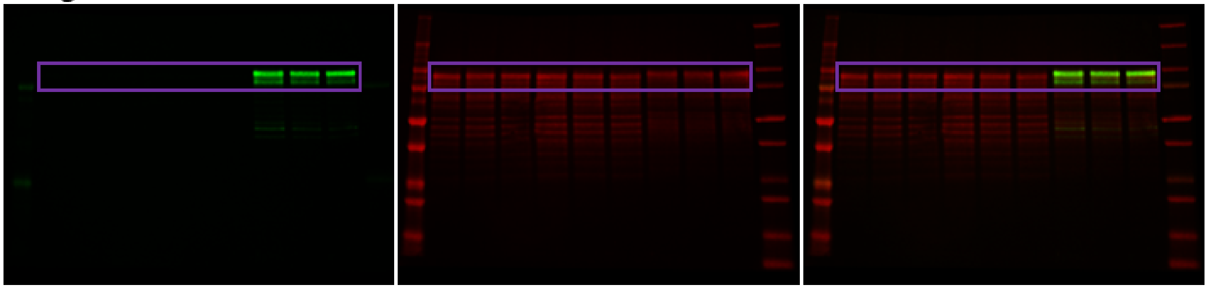

Fig. S7c

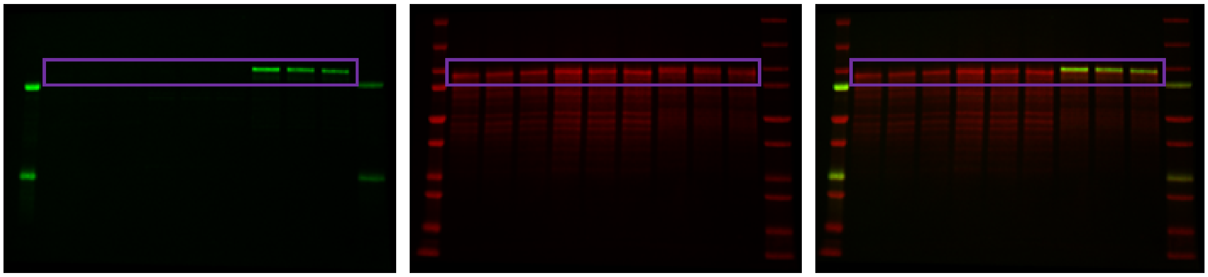

Additional References

[1] A. E. McEwen, M. T. Maher, R. Mo, C. J. Gottardi, *Mol. Biol. Cell* **2014**, *25*, 2365-2374.

[2] H. d. Santos Seckler, H.-M. Park, C. M. Lloyd-Jones, R. D. Melani, J. M. Camarillo, J. T. Wilkins, P. D. Compton, N. L. Kelleher, *Journal of the American Society for Mass Spectrometry* **2021**, *32*, 1659-1670.

[3] J. P. McGee, M. W. Senko, K. Jooß, B. J. Des Soye, P. D. Compton, N. L. Kelleher, J. O. Kafader, *Anal. Chem.* **2022**, *94*, 16543-16548.

[4] P. Su, J. P. McGee, M. A. R. Hollas, R. T. Fellers, K. R. Durbin, J. B. Greer, B. P. Early, P. F. Yip, V. Zabrouskov, K. Srzentić, M. W. Senko, P. D. Compton, N. L. Kelleher, J. O. Kafader, *Nat. Protoc.* **2025**, *20*, 1485-1508.

[5] L. Fornelli, K. Srzentić, R. Huguet, C. Mullen, S. Sharma, V. Zabrouskov, R. T. Fellers, K. R. Durbin, P. D. Compton, N. L. Kelleher, *Anal. Chem.* **2018**, *90*, 8421-8429.

[6] C.-F. Huang, J. T. Kline, F. Negrão, M. T. Robey, T. K. Toby, K. R. Durbin, R. T. Fellers, J. J. Friedewald, J. Levitsky, M. M. I. Abecassis, R. D. Melani, N. L. Kelleher, L. Fornelli, *Anal. Chem.* **2024**, *96*, 3578-3586.

[7] D. N. Perkins, D. J. C. Pappin, D. M. Creasy, J. S. Cottrell, *Electrophoresis* **1999**, *20*, 3551-3567.

[8] P. Mertins, F. Yang, T. Liu, D. R. Mani, V. A. Petyuk, M. A. Gillette, K. R. Clauser, J. W. Qiao, M. A. Gritsenko, R. J. Moore, D. A. Levine, R. Townsend, P. Erdmann-Gilmore, J. E. Snider, S. R. Davies, K. V. Ruggles, D. Fenyo, R. T. Kitchens, S. Li, N. Olvera, F. Dao, H. Rodriguez, D. W. Chan, D. Liebler, F. White, K. D. Rodland, G. B. Mills, R. D. Smith, A. G. Paulovich, M. Ellis, S. A. Carr, *Mol. Cell. Proteomics* **2014**, *13*, 1690-1704.
